# Six-month performance and safety of an iris-fixated multifocal intraocular lens for presbyopia correction in phakic eyes

**DOI:** 10.1097/j.jcrs.0000000000001722

**Published:** 2025-10-23

**Authors:** Joukje C. Wanten, Julián Cezón, Rudy M.M.A. Nuijts, Juan A. Duran de la Colina, Mariano Royo, Chul Myong Choe, Jae Ho Choi, Chan Soo Park, Il Hwan Koh, Daniel Casado Rodríguez, Noël J.C. Bauer, José L. Güell

**Affiliations:** From the University Eye Clinic, Maastricht University Medical Center, Maastricht, the Netherlands (Wanten, Nuijts, Bauer); Clinica CIMO, Seville, Spain (Cezón); ICQO Instituto Clínico Quirúrgico de Oftalmologia, Bilbao, Spain (Duran de la Colina); Hospital San Rafael, Madrid, Spain (Royo); Nune Eye Hospital, Daegu, South Korea (Choe, Choi); GM St. Mary's Eye Clinic, Busan, South Korea (Park); SU Yonsei Eye Clinic, Seoul, South Korea (Koh); Visiondiez, Madrid, Spain (Rodríguez); IMO Instituto de Microcirugía Ocular, Barcelona, Spain (Güell).

## Abstract

**Purpose:**

To evaluate the 6-month effectiveness and safety of a new foldable multifocal iris-fixated phakic intraocular lens (pIOL) for presbyopia correction.

**Setting:**

Multicenter study at 7 sites.

**Design:**

Nonrandomized prospective clinical trial.

**Methods:**

Presbyopic patients without cataracts undergoing bilateral multifocal pIOL implantation (Ophtec ArtiPlus) were studied. The primary outcome for efficacy was the visual acuity at distance, intermediate, and near. Secondary outcomes included endothelial cell density (ECD), postoperative refraction, binocular defocus curve, vision quality, spectacle independence, and patient satisfaction.

**Results:**

49 subjects (98 eyes) were included. At 6 months, the mean binocular uncorrected distance intermediate, and near visual acuity was −0.05 ± 0.09 logMAR, −0.02 ± 0.07 logMAR, and 0.02 ± 0.08 logMAR, respectively. The ECD remained stable with a mean value of 2771 ± 289 cells/mm^2^ at 6 months postoperatively. The mean spherical equivalent was −0.42 ± 0.33 diopters (D), with 95% eyes having a prediction error within ±1.0 D and 77% within ±0.5 D. The binocular distance-corrected defocus curve showed a visual acuity of 0.20 logMAR or better over a range of +1.0 to −3.5 D. Patient satisfaction was high, with a mean score of 3.5 on a 4-point scale, and 83% of the patients reported spectacle independence. 47%, 35%, and 96% of patients reported experiencing glare, halos, and starbursts at well-tolerated levels, respectively. Notably, only 2% of patients found halos and starbursts to be very bothersome.

**Conclusions:**

Bilateral ArtiPlus multifocal pIOL implantation demonstrated good visual acuity from distant to near and maintained stable ECD over 6 months. Patients reported high levels of satisfaction and spectacle independence, despite some optical disturbances.

## Abstract 2

The implantation of this new multifocal pIOL proves to be effective and safe and provides middle-aged presbyopia patients a high level of spectacle independence and satisfaction.

In the past few decades, various innovative technologies have been introduced in refractive surgery to address the needs of presbyopic patients seeking spectacle independence. As the prevalence of this age-related condition increases and its associated spectacle dependence significantly reduces quality of life, more presbyopic patients are seeking freedom from corrective eyewear.^1,2^ Presbyopia correction is considered the last significant challenge of refractive surgery, with a range of surgical techniques available, including both cornea-based and lens-based procedures. No single technique has emerged as superior because patient suitability varies based on specific characteristics. Among the lens-based treatment options are refractive lens exchange with multifocal intraocular lens (mIOL) implantation or multifocal phakic intraocular lens (pIOL) implantation.^2–4^ The use of mIOLs has proven effective for achieving complete spectacle independence.^5,6^ Multifocal pIOL implantation offers a promising alternative. It combines the benefits of reversibility with a lower risk of retinal complications, making it particularly suitable for middle-aged, presbyopic patients.^2,5,6^ Previous studies have demonstrated the safety and efficacy of pIOLs in correcting myopia, with outcomes indicating high levels of patient satisfaction and visual acuity.^4,7^ The new multifocal ArtiPlus IOL integrates the platform of the Artiflex Myopia lens with the optical design of the Precizon Presbyopic IOLs, both previously developed by Ophtec BV, Groningen, the Netherlands. The purpose of this study was to evaluate the effectiveness and safety of the new multifocal ArtiPlus pIOL in presbyopic adults desiring spectacle independence for all distances.

## METHODS

This study was a multicenter, prospective clinical trial conducted at 4 hospitals in Spain (IMO Institutio de Microcirurgía Ocular, Barcelona; Clinica CIMO, Seville; ICQO Instituto Clínico Quirúgico de Oftalmologia, Bilbao; and Servicio de Oftalmologia San Rafael, Madrid) and 3 hospitals in South Korea (Nune Eye Hospital, Daegu; GM St. Mary's Eye Clinic, Busan; and SU Yonsei Eye Clinic, Seoul). Participants were enrolled from each center. Presbyopic adults without cataract, seeking spectacle independence for distant to near vision, were included if they had a stable refractive spherical equivalent (SE) of ±0.75 diopters (D) for at least 12 months before surgery, and an expected residual postoperative cylindrical refractive error ≤0.75 D and an expected postoperative corrected distance visual acuity (CDVA) of ≤0.2 logMAR. Patients were excluded if the selection criteria for pIOL implantation were not met according to the instructions for use. Full details about the selection criteria are summarized in Appendix 1 (available at http://links.lww.com/JRS/B389). All patients signed informed consent before enrollment. This study was approved by the Medical Ethics Committee at IMO and “Agencia Española de Medicamentos y Productos Sanitarios (AEMPS)” in Spain, and the local ethics committee at each site in South Korea. The Korean competent authority, MFDS, also assessed and approved the study. This study adhered to the tenets of the Declaration of Helsinki. The trial was registered at ClinicalTrial.gov (NCT04632784).

**Figure 1.**
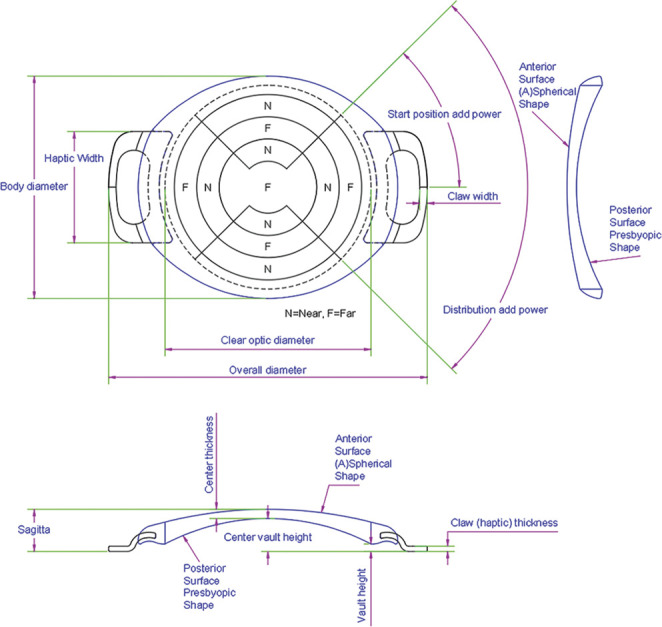
The Artiflex Presbyopic IOL design with distribution of the far/near segments. F = far; N = near.

Patients were scheduled for bilateral ArtiPlus (Ophtec BV) pIOL implantation. The ArtiPlus is a foldable anterior chamber iris-fixated multifocal pIOL made of polysiloxane, with a diopter range between −15.0 D and +2.0 D and an add-on power of +2.5 D for near vision. This pIOL features an aspheric segmented optic designed with multiple elongated focal points, enabling a seamless transition between distance and near vision. The design includes 5 segments for far vision and 6 segments for near vision, as illustrated in Figure 1. Lens power calculation was performed by Ophtec BV (Groningen, the Netherlands) using the modified Van der Heijde formula targeted for emmetropia.^8^ Surgeries were performed by experienced surgeons using a 3.2 mm main incision positioned at 90 degrees from the intended enclavation axis. The pIOL was implanted and fixated to the iris. An iridectomy or iridotomy was performed either during surgery or at least 1 week before pIOL implantation using a Nd:YAG laser. If necessary, a suture was used for wound closure. The surgeries for the first and second eye were performed within a 2-week interval.

Patients underwent preoperative testing, including manifest subjective and cycloplegic refraction, visual acuity assessments, biometry (IOLMaster, Carl Zeiss Meditec AG; Alladin, Topcon Corp.; Argos, Alcon Laboratories, Inc.), corneal topography and tomography (Pentacam, Oculus Optikgeräte GmbH; Alladin, Topcon Corp,; Gallilei G4, Ziemer Ophthalmic Systems AG), pupillometry, tonometry, gonioscopy, specular microscopy, contrast sensitivity testing (CSV-1000, Vector Vision, Inc.), and slitlamp and funduscopic examination. Postoperative assessments were performed at 1 day, 1 week, 1 month, 3 months, and 6 months after pIOL implantation. Visual acuity assessments were conducted using the Early Treatment Diabetic Retinopathy Study (ETDRS) charts at 4 m, 80 cm, and 40 cm for distant, intermediate, and near vision, respectively. The logMAR score was determined by identifying the last attempted line on the ETDRS chart until no further optotypes could be distinguished. This score was obtained by adding the total number of correctly identified optotypes to the score of the last attempted line. A binocular corrected defocus curve was performed 6 months postoperatively under photopic conditions, with a defocus range of +1.5 to −5.0 D with 0.50 D increments. Preoperatively and at 6 months postoperatively, the quality of vision (QoV) was evaluated using the validated QoV questionnaire.^9^ Patient satisfaction was assessed at 6 months postoperatively through an unvalidated satisfaction questionnaire which can be found in Appendix 2 (available at http://links.lww.com/JRS/B390). Both questionnaires use 4-point Likert scales, ranging from zero (representing none/never) to 4 (indicating severe/always).

### Statistical Analyses

Data analysis was conducted using SPSS (IBM SPSS Statistics for Windows, v. 28.0, Released 2021, IBM Corp.). All qualitative variables were summarized as the distribution of frequency, absolute, and percentage. For all quantitative variables, descriptive statistics were calculated. Statistical comparison of endothelial cell density (ECD) and intraocular pressure (IOP) over time was calculated using the analysis of variance–repeated measures in case of normality. In case of nonnormally distributed data, nonparametric tests were used. Comparisons between preoperative and postoperative QoV questionnaires were performed using the Wilcoxon signed-rank test. Associations between optical side effects and pupillary sizes were tested using ordinal regression analysis. A significance level of ≤0.05 was applied.

## RESULTS

From November 2021 to March 2023, a total of 49 patients (98 eyes) were enrolled in the study. The cohort consisted of 22 men (45%) and 27 women (55%), with a mean age of 49 ± 5 years (range: 43 to 64). The ethnic distribution included 21 White patients (43%) and 28 Asian patients (57%). Table 1 presents the baseline characteristics. Note that 1 patient missed the 6-month postoperative visit.

**Table 1.** Baseline characteristics of 49 patients (98 eyes)

Parameter	Mean ± SD (range)
Refraction (manifest)	
Sphere (D)	−4.64 ± 2.23 (−10.50, 1.75)
Cylinder magnitude (D)	−0.68 ± 0.42 (−2.25, 0.00)
SE (D)	−4.98 ± 2.27 (−11.00, 1.50)
Refraction (cycloplegic)	
Sphere (D)	−4.55 ± 2.26 (−9.75, 2.00)
Cylinder magnitude (D)	−0.67 ± 0.44 (−2.50, 0.00)
SE (D)	−4.88 ± 2.30 (−10.37, 1.75)
CDVA, monocular (logMAR)	0.00 ± 0.08 (−0.20, 0.40)
IOP (mm Hg)	15.0 ± 3.1 (9.0, 21.0)
AL (mm)	25.68 ± 1.01 (23.36, 28.69)
ACD (mm)	3.24 ± 0.26 (2.83, 3.83)
Keratometry (D)	44.23 ± 1.22 (41.38, 46.85)
ECD (cells/mm^2^)	2785 ± 238 (2057, 3436)
Pupillometry	
Photopic (mm)	3.79 ± 0.76 (2.75, 6.20)
Mesopic (mm)	5.51 ± 1.02 (3.30, 7.00)
Scotopic (mm)	6.11 ± 0.83 (3.10, 7.00)
IOL power (D)	−5.53 ± 2.42 (−11.50, 2.00)
Target (D)	−0.07 ± 0.18 (−0.95, 0.28)

ACD = anterior chamber depth; AL = axial length; CDVA = corrected distance visual acuity; D = diopter; ECD = endothelial cell density; IOL = intraocular lens; IOP = intraocular pressure; SD = standard deviation; SE = spherical equivalent

### Short-Term Efficacy

**Figure 2.**
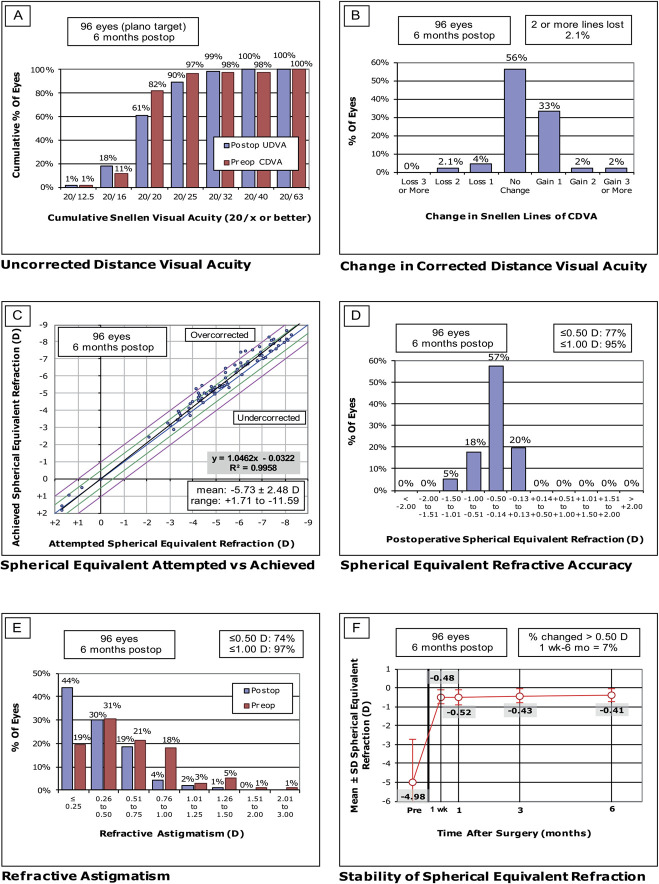
Visual and refractive outcomes after bilateral implantation of the ArtiPlus phakic IOL. A: UDVA outcomes. B: Postoperative UDVA and preoperative CDVA. C: Distribution of achieved spherical equivalent outcomes. The green lines represent outcomes within ±0.50 D and the pink lines within ±1.0 D. D: Spherical equivalent refractive accuracy. E: Refractive astigmatism. F: Spherical equivalent refraction stability. CDVA = corrected distance visual acuity; D = diopter; UDVA = uncorrected distance visual acuity.

**Figure 3.**
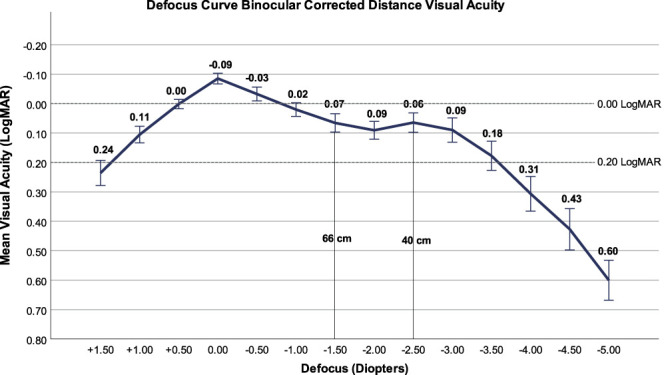
Binocular distance-corrected visual acuity defocus curve (n = 48).

The monocular refractive and visual outcomes at 1, 3, and 6 months postoperatively are detailed in Table 2. The mean uncorrected and distance-corrected binocular outcomes at 6 months postoperatively were −0.05 ± 0.09 and −0.09 ± 0.06 logMAR for distance (uncorrected distance visual acuity [UDVA] and CDVA), −0.02 ± 0.07 and 0.00 ± 0.09 logMAR for intermediate (uncorrected intermediate visual acuity [UIVA] and distance corrected intermediate visual acuity), and 0.02 ± 0.08 and 0.01 ± 0.06 logMAR for near vision (uncorrected near visual acuity [UNVA] and distance corrected near visual acuity), respectively. The binocular corrected near visual acuity was −0.02 ± 0.05 logMAR. The efficacy indices at 1 month, 3 months, and 6 months (mean postoperative UDVA/mean preoperative CDVA) were 0.81, 0.91, and 0.99, respectively. The postoperative uncorrected and preoperative corrected visual outcomes for all eyes are displayed in Figure 2A. Figure 3 shows the binocular distance corrected binocular defocus curve at 6 months postoperatively (n = 48). The visual acuity was 0.20 logMAR or better over a range of +1.0 to −3.5 D.

**Table 2.** Monocular refractive and visual acuity outcomes at multiple timepoints

Parameter	Preop (n = 98)	1 mo postop (n = 98)	3 mo postop (n = 98)	6 mo postop (n = 96)
Sphere (D)	−4.64 ± 2.23	−0.27 ± 0.36	−0.22 ± 0.31	−0.21 ± 0.29
Cylinder magnitude (D)	−0.68 ± 0.42	−0.50 ± 0.38	−0.44 ± 0.35	−0.41 ± 0.35
SE (D)	−4.98 ± 2.27	−0.52 ± 0.40	−0.43 ± 0.35	−0.41 ± 0.33
UDVA (logMAR)	0.94 ± 0.25	0.09 ± 0.15	0.04 ± 0.11	0.02 ± 0.09
CDVA (logMAR)	0.00 ± 0.08	0.00 ± 0.10	−0.03 ± 0.07	−0.05 ± 0.06
UIVA (logMAR)	0.73 ± 0.36		0.04 ± 0.10	0.02 ± 0.08
DCIVA (logMAR)	0.08 ± 0.14		0.05 ± 0.12	0.03 ± 0.10
UNVA (logMAR)	0.47 ± 0.42	0.11 ± 0.16	0.10 ± 0.12	0.07 ± 0.09
DCNVA (logMAR)	0.15 ± 0.21		0.10 ± 0.13	0.04 ± 0.08
CNVA (logMAR)	0.01 ± 0.07		0.02 ± 0.06	0.01 ± 0.06

CDVA = corrected cistance visual acuity; CNVA = corrected near visual acuity; D = diopters; DCIVA = distance-corrected intermediate visual acuity; DCNVA = distance-corrected near visual acuity; SD = standard deviation; SE = spherical equivalent; UDVA = uncorrected distance visual acuity; UIVA = uncorrected intermediate visual acuity; UNVA = uncorrected near visual acuity

### Short-Term Safety

The safety assessment focused on ECD, with mean values of 2760 ± 225 cells/mm^2^ and 2809 ± 249 cells/mm^2^ preoperatively, 2753 ± 265 cells/mm^2^ and 2786 ± 268 cells/mm^2^ at 1 month, and 2750 ± 271 cells/mm^2^ and 2785 ± 309 cells/mm^2^ at 6 months postoperatively, for the right and left eyes, respectively. The ECD remained stable over the first 6 months postoperatively, with no significant differences observed over time for both the right (*P* = .858) and left (*P* = .344) eyes. At the 6-month follow-up, the IOP had a mean value of 14.2 ± 2.6 mm Hg for the right eyes and 14.2 ± 2.8 mm Hg for the left eyes. No significant differences were found in IOP over time for the right (*P* = .308) and left (*P* = .217) eyes. The safety indices (ratio of mean postoperative CDVA to mean preoperative CDVA) at 1 month, 3 months, and 6 months were 1.00, 1.07, and 1.12, respectively. There was a gain or no change in Snellen lines of CDVA for 94% of the eyes at 6 months postoperatively and 2.1% lost 2 or more lines, displayed in Figure 2B.

### Predictability and Stability

The mean prediction error was −0.42 ± 0.33 D at 6 months postoperatively. The SE of 77% of eyes was within ±0.5 D and 95% of eyes within ±1.0 D from the intended target. The mean refractive cylinder magnitude was −0.41 ± 0.35 D, with 74% eyes showing a residual cylinder of ≤0.50 D and 97% of ≤1.00 D. Predictability outcomes are displayed in Figure 2C–E. The SE remained stable over 6 months, with 7% of the eyes exhibiting a change >0.50 D between week 1 and 6 months, as visualized in Figure 2F.

### Complications and Adverse Events

One month postoperatively, one patient (1 eye) was diagnosed with non–lens-related optic neuritis, while another patient (both eyes) had elevated IOP because of corticosteroid treatment occurred. In addition, a third patient (both eyes) had developed cell deposits in the anterior chamber. At 3 months postoperatively, one patient (1 eye) had retained cell deposits, and another patient (1 eye) was diagnosed with viral conjunctivitis. No other adverse events were reported.

### Quality of Vision

**Figure 4.**
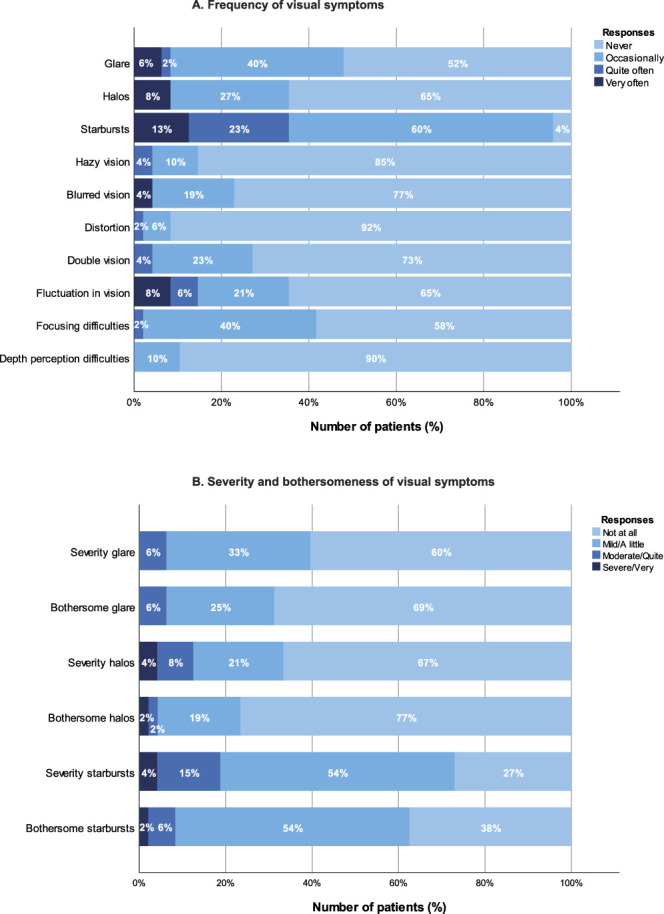
Outcomes QoV questionnaire at 6 months postoperatively (n = 48), including (A) the frequency of visual symptoms and (B) the severity and bothersomeness of the visual symptoms. QoV = quality of vision.

The postoperative contrast sensitivity (n = 31) was comparable in photopic and mesopic conditions, with and without glare. These contrast sensitivities were within normal limits and are presented in Appendix 3 (available at http://links.lww.com/JRS/B391). The postoperative outcomes of the QoVquestionnaire can be found in Figure 4A and B. Most patients experienced never or only occasionally glare or halos, although starburst was more frequently reported. Starbursts were reported as little bothersome in 54%, quite bothersome in 6%, and very bothersome in 2% of the patients. Overall, visual symptoms remained unchanged for 50% of the patients when compared with the preoperative assessments, improved for 48% and worsened for 2%. There was no difference found between the preoperative and postoperative existence of glare and halos, but for the presence of starbursts, there was a significant increase found with a Z-score of 3.26 (*P* = .001). Preoperative pupil size under photopic, mesopic, or scotopic light conditions did not show a significant association with postoperative optical side effects.

### Spectacle Independence and Patient Satisfaction

Six months postoperatively, most of the patients (98%) reported to be quite or very satisfied, rating 3 or 4 on a 4-point Likert scale, respectively. One patient reported little satisfaction with the results due to continued spectacle dependence. All patients were satisfied with their distant vision and intermediate vision, except for 1 patient who expressed only slight satisfaction with intermediate vision. For near vision, 5 patients indicated little satisfaction (rating 2 on a 4-point Likert scale), while the majority reported being satisfied. In total, 98% of the patients would choose the same procedure again. Regarding spectacle independence, 83% (n = 40) reported never using spectacles or contact lenses, 15% (n = 7) used them occasionally, and 1 patient reported to use them very often. Those patients who did require spectacles primarily needed them for far and near distances.

## DISCUSSION

To the authors' knowledge, this multicenter prospective trial is the first study to report on the 6-month postoperative performance and safety after bilateral implantation of the iris-fixated multifocal ArtiPlus pIOL. The results demonstrated that the ArtiPlus pIOL is an effective treatment for correcting presbyopia in adult patients without cataract who seek spectacle independence. The study showed predictable and stable outcomes over the first 6 postoperative months. Safety indices were high, and the ECD remained stable throughout this period. In addition, most of the patients reported high satisfaction with the results, with 83% of patients achieving full spectacle independence.

Previous studies have demonstrated that the implantation of iris-fixated pIOLs (Artiflex pIOL) is effective and safe to correct patients with myopia with or without myopic astigmatism.^10–15^ The ArtiPlus pIOL is the only anterior chamber, iris-fixated presbyopia-correcting pIOL which is currently available. In this study, the mean binocular UDVA, UIVA, and UNVA at 6 months were excellent, with values of −0.05 ± 0.09 logMAR, −0.02 ± 0.07 logMAR, and 0.02 ± 0.08 logMAR, respectively. Although all eyes were targeted for emmetropia, the refractive outcomes were slightly more myopic, with a mean SE of −0.41 ± 0.33 D. This myopic outcome may have contributed to enhanced visual acuity at intermediate and near distances. At 6 months, the efficacy index was 0.99 and the binocular corrected defocus curve showed a mean visual acuity better than 0.2 logMAR across a broad defocus range from +1.0 to −3.50 D. These findings are consistent with the reported visual acuity outcomes for distance and efficacy indices after monofocal Artiflex pIOL implantations.^13,14,16^

Other currently available presbyopia-correcting pIOLs include posterior chamber pIOLs—implantable collamer lenses (ICLs) or implantable phakic contact lenses (IPCLs). However, only limited and short-term data are reported in the literature. The EVO Viva ICL (STAAR Surgical Co.), designed to extend depth of focus by 2.0 D, has demonstrated a mean binocular UDVA and CDVA of 0.09 ± 0.09 logMAR and 0.02 ± 0.03 logMAR after an average follow-up of 8 months. The corresponding SE is −0.61 ± 0.54 D, with 60% of the eyes achieving a refractive outcome within ±0.50 D and 87.5% within ±1.00 D of target. The binocular distance-corrected defocus curve shows a visual acuity ≤0.2 logMAR between +2.0 D and −2.0 D of defocus. In addition, the efficacy and safety indices of this ICL are 0.78 and 0.97, respectively.^17^ Another EVO + Visian ICL (STAAR Surgical Co.) study with 34 patients, also designed to provide up to 2.0 D of extended depth of focus, demonstrates a 6-month postoperative binocular UDVA, UIVA, and UNVA of 0.07 ± 0.10 logMAR, −0.01 ± 0.05 logMAR, and −0.02 ± 0.08 logMAR, respectively. The binocular distance-corrected defocus curve shows a visual acuity of ≤0.2 logMAR between +1.5 D and −2.5 D of defocus. Most of the patients (91.2%) report being overall satisfied, while there is also a vision-related decrease in quality of life due to glare symptoms.^18^ Furthermore, 2 small studies investigating the presbyopia IPCL (Care Group India), available with additions from +1.5 to +4.0 D report a postoperative SE of −0.25 ± 0.25 D at 12 months postoperatively. The binocular uncorrected defocus curve demonstrates a visual acuity of ≤0.2 logMAR between +0.5 D and −3.5 D of defocus.^19,20^ Overall, compared with these other available multifocal pIOLs, the ArtiPlus pIOL showed superior visual acuity results.

In this patient cohort, optical disturbances were commonly reported, with 47.9% of patients experiencing glare, 35.4% experiencing halos, and 95.8% experiencing starbursts; however, only 8.3% found these disturbances to be bothersome. A previous study also reported high rates of optical disturbances related to night vision in patients after Artisan pIOL implantation, with halos and starbursts occurring in 48.8% and starbursts in 41.7% of cases. Furthermore, the study highlights that glare associated with this pIOL varies under different weather conditions, with prevalence rates of 50.8% on sunny days, 42.6% in snowy weather conditions, and 12.5% in foggy weather.^15^ Other studies investigating the Artisan pIOL report lower incidence of halos and glare, ranging between 6.0% and 8.0%.^21,22^ Research involving the Artiflex pIOL reports an incidence of mild to moderate glare of 7.0% to 13.9% and of mild halos in 4.3% to 11.3% during the early postoperative period.^23^ Furthermore, a comparative study concludes that there is no significant difference in incidence of halos and glare between Artisan and Artiflex pIOLs.^24^

Our results demonstrated a higher incidence of starbursts, which were reported by nearly all patients. These discrepancies with the existing literature may be attributed to the fact that multifocal lens designs generally carry a higher risk of optical side effects compared with monofocal lens designs.^25^ In this cohort, no association with preoperative pupillary size was found to further explain the high incidence of these optical disturbances, and data regarding aberrometry and stray light have not been collected. Literature suggests that, in general, complaints of positive dysphotopsia may be a result of insufficient neuroadaptation after IOL implantation.^26,27^ In our findings, patients who experienced bothersome starbursts often also reported bothersome halos and glare, which could be a result of an insufficient neuroadaptation at 6 months postoperatively. Further long-term studies are necessary to clarify the extent and mechanisms of these optical side effects in patients with multifocal pIOLs.

Although optical disturbances were reported at relatively high rates, they were bothersome to only a small percentage of patients, with the majority (98%) expressing satisfaction with the results. Only 1 patient reported little satisfaction, which was attributed to bothersome glare, halos, and starbursts, as well as continued spectacle dependence for distant vision. Overall, the spectacle independence after bilateral ArtiPlus implantation was high at all distances, with 83% of the patients being fully spectacle independent for all ranges of vision.

Over the course of 6 months, the ArtiPlus pIOL showed safe and stable results. However, no conclusions can yet be drawn regarding long-term outcomes. The ECD remained stable during this period, but a longer follow-up period is required to assess the long-term impact on ECD. The mean reported survival time of a pIOL is 10 to 15 years, with ECD loss and cataract formation being the primary reasons for explantation.^28–31^ Studies on long-term ECD loss after Artiflex pIOL implantation report an average ECD loss of 0.98% to 2.0% each year.^14,16,32^ Generally, these monofocal pIOLs are implanted in relatively young, healthy patients. The population of this study is older than 40 years of age, which reduces the long-term risks, because the time to cataract surgery is shorter compared with nonpresbyopic patients receiving pIOLs.^3^ Despite this, strict long-term follow-up, including annual ECD monitoring, is recommended to detect ECD loss at an early stage.^28,30^ In addition, further research is necessary to evaluate the additional risk of cataract formation in this population.

Limitations of this study were the small sample size and short follow-up time, and therefore, additional research is essential to investigate the long-term outcomes of this new multifocal pIOL. In conclusion, this study demonstrated that binocular implantation of ArtiPlus pIOLs is a safe and effective approach, providing excellent vision across all distances 6 months postoperatively. This new pIOL design enabled presbyopic patients to achieve spectacle independence, with high overall satisfaction and an acceptable level of bothersome optical disturbances.

**WHAT WAS KNOWN**

- Implantation of foldable iris-fixated pIOLs has been proven safe and effective for myopia correction, with or without myopic astigmatism.
- Presbyopia correction remains a significant challenge in refractive surgery, with both cornea-based and lens-based techniques available. No single method is universally superior due to patient-specific factors.

**WHAT THIS PAPER ADDS**

- The new multifocal iris-fixated pIOL (ArtiPlus) effectively corrects presbyopia in middle-aged patients, achieving high spectacle independence and patient satisfaction.
- Optical disturbances were reported but were generally non-bothersome.
- This study demonstrated that the multifocal pIOL yields safe, predictable, and stable outcomes up to 6 months postoperatively.
